# Academic Outcomes and Utility of Gastroenterology Residents In-Training Course Abstract Presentations: The University of British Columbia Experience

**DOI:** 10.1155/2016/8698632

**Published:** 2016-10-23

**Authors:** Faisal Al Shatti, Vladimir Marquez Azalagara, Eric M. Yoshida

**Affiliations:** Division of Gastroenterology, University of British Columbia, Vancouver, BC, Canada

The Gastroenterology Residents-in-Training (GRIT) course is held in conjunction with the annual Canadian Digestive Disease Week. The format of the GRIT course requires Gastroenterology trainees to submit an abstract, and if accepted, the abstract is presented. At UBC, it is strongly recommended that trainees submit to the meeting.

A questionnaire composed of 11 multiple choice questions was sent to all former and current trainees. The aim was to assess the utility of the GRIT course from a UBC academic perspective by reviewing the outcomes (including publication and presentation at international meetings) of the projects submitted and to determine the value of the process to the trainees. 88.8% of fellows responded (32 of 36). 43.75% are currently working in academic centers, 37.5% are in the community, and 18.75% are still in training (that may be extra to core GI training). The GRIT abstract was a case report (33.3%), a clinical research (61.9%), or a basic science project (4.8%). 43.75% were presented at international meetings. 68.75% were published (only one was a nonpeer review paper). The reasons for not publishing were “too busy and not enough time given during my training” (22.2%), “the abstract was appropriate for the GRIT/CDDW meeting, I did not feel that it was strong enough to be published in a journal” (44.5%), and “the abstract reported work that was part of a greater research project and I was not significantly involved in the overall project” (33.3%). 21.8% received awards for their projects either at the GRIT course or at UBC trainee research days. 68.3% thought the GRIT experience was worthwhile, although one responder thought it was irrelevant.

To the best of our knowledge there are only two papers published in Canadian literature that have looked at the publication rates of trainee research projects.

Gill et al. [1] in 2001 reported a UBC medical resident research day conversion to publication rates of 28%. Hung and Duffett [2], reporting Canadian pharmacy resident conversion rates, found only 32.3% of resident projects became publications and only 20.6% became full papers.

Although the sample size is comparatively small, our GRIT course experience appears to differ as more than two-thirds of the projects were published. Importantly, most responders thought that the GRIT experience was worthwhile. We speculate that there are many reasons why the publication rate was higher in our GI trainees. The trainees, being older than those studied by Gill et al. [1] and Hung and Duffett [2], may have been more motivated because of pressure to improve CVs and obtain faculty positons after training. The faculty may also have been more motivated to “push” the trainees to complete and publish the research project. Regardless, at UBC, it appears that the GRIT course has been a successful and rewarding experience for trainees.
